# Neuromodulatory effects and reproducibility of the most widely used repetitive transcranial magnetic stimulation protocols

**DOI:** 10.1371/journal.pone.0286465

**Published:** 2023-06-23

**Authors:** Justine Magnuson, Mehmet A. Ozdemir, Elon Mathieson, Sofia Kirkman, Brice Passera, Sumientra Rampersad, Alyssa B. Dufour, Dana Brooks, Alvaro Pascual-Leone, Peter J. Fried, Mouhsin M. Shafi, Recep A. Ozdemir

**Affiliations:** 1 Berenson-Allen Center for Noninvasive Brain Stimulation, Department of Neurology, Beth Israel Deaconess Medical Center, Boston, MA, United States of America; 2 Department of Neurology, Harvard Medical School, Boston, MA, United States of America; 3 Health and Exercise Sciences, University of British Columbia Okanagan, Kelowna, BC, CA; 4 Department of Biomedical Engineering, Izmir Katip Celebi University, Izmir, Turkey; 5 Department of Physics, University of Massachusetts, Boston, MA, United States of America; 6 Department of Electrical and Computer Engineering, Northeastern University, Boston, MA, United States of America; 7 Department of Medicine, Beth Israel Deaconess Medical Center, Harvard Medical School, Boston, MA, United States of America; 8 Hinda and Arthur Marcus Institute for Aging Research, Hebrew Senior Life, Boston, MA, United States of America; 9 Hinda and Arthur Marcus Institute for Aging Research and Deanne and Sidney Wolk Center for Memory Health, Hebrew SeniorLife, Boston, MA, United States of America; 10 Guttmann Brain Health Institute, Institut Guttmann de Neurorehabilitació, Universitat Autonoma de Barcelona, Badalona, Spain; University of Ottawa, CANADA

## Abstract

**Background:**

Repetitive transcranial magnetic stimulation (rTMS) is widely used in both research and clinical settings to modulate human brain function and behavior through the engagement of the mechanisms of plasticity. Based upon experiments using single-pulse TMS as a probe, the physiologic mechanism of these effects is often assumed to be via changes in cortical excitability, with 10 Hz rTMS increasing and 1 Hz rTMS decreasing the excitability of the stimulated region. However, the reliability and reproducibility of these rTMS protocols on cortical excitability across and within individual subjects, particularly in comparison to robust sham stimulation, have not been systematically examined.

**Objectives:**

In a cohort of 28 subjects (39 ± 16 years), we report the first comprehensive study to (1) assess the neuromodulatory effects of traditional 1 Hz and 10 Hz rTMS on corticospinal excitability against both a robust sham control, and two other widely used patterned rTMS protocols (intermittent theta burst stimulation, iTBS; and continuous theta burst stimulation, cTBS), and (2) determine the reproducibility of all rTMS protocols across identical repeat sessions.

**Results:**

At the group level, neither 1 Hz nor 10 Hz rTMS significantly modulated corticospinal excitability. 1 Hz and 10 Hz rTMS were also not significantly different from sham and both TBS protocols. Reproducibility was poor for all rTMS protocols except for sham. Importantly, none of the real rTMS and TBS protocols demonstrated greater neuromodulatory effects or reproducibility after controlling for potential experimental factors including baseline corticospinal excitability, TMS coil deviation and the number of individual MEP trials.

**Conclusions:**

These results call into question the effectiveness and reproducibility of widely used rTMS techniques for modulating corticospinal excitability, and suggest the need for a fundamental rethinking regarding the potential mechanisms by which rTMS affects brain function and behavior in humans.

## Introduction

Over the past several decades, repetitive transcranial magnetic stimulation (rTMS) has become the most widely used non-invasive technique for modifying human brain function in both health and disease. Conventional rTMS protocols consist of repeated trains of pulses delivered at low (1 Hz) or high frequencies (≥ 10 Hz) [1, 2], with the goal of suppressing (inhibitory) or facilitating (excitatory) neural excitability, respectively [3–5]. More recently, adopted from animal studies, accelerated high-frequency rTMS protocols called theta burst stimulation (TBS) have been developed to induce similar inhibitory and excitatory neuromodulatory effects [6]. These TBS protocols have received increased attention in recent years owing to their shorter durations and low stimulation intensities. The widespread use of all rTMS protocols in both clinical and experimental research is motivated by the assumption that they can induce lasting and reliable changes in neuronal function (e.g., excitability) in the stimulated brain region (e.g., motor cortex) and its connected circuits, and thus modulate corresponding behaviors and/or cortical responses (e.g., motor-evoked potentials, MEPs).

While initial studies reported expected neuromodulatory effects [6, 7] and partial reproducibility of certain rTMS protocols [8], growing evidence consistently suggests that neuromodulatory effects of rTMS are highly variable across subjects, protocols and sessions. Recent work has extensively focused on the effectiveness and reproducibility of TBS protocols and reported both high inter- and intra-individual response variability with poor reproducibility across repeat TBS sessions [9–16]. Surprisingly, however, the only study assessing intra-individual reliability of traditional 1 Hz and 10 Hz rTMS protocols was reported more than two decades ago [8] in a relatively smaller sample size (*n* = 20), with no delayed post-rTMS measurements, on consecutive days when meta-plasticity effects might be present, and, most importantly, without having a robust sham rTMS control. Our recent work using intermittent and continuous TBS protocols demonstrated that most of the significant TBS aftereffects on cortical and corticospinal responses were not statistically different from sham TBS [12]. More importantly, none of the significant neuromodulatory effects of TBS were reproducible across identical sessions [12]. These results endorse sham controls as an essential part of any rTMS design and suggest that the neuromodulatory effects and reproducibility of the two most widely used clinical rTMS protocols (1 Hz and 10 Hz) need to be validated against sham stimulation.

Given the lack of sham-controlled studies assessing reproducibility of 1 Hz and 10 Hz rTMS protocols, we performed a comprehensive study to (1) assess the neuromodulatory effects of traditional low- and high-frequency rTMS protocols against a robust sham control, (2) compare their neuromodulatory effects and reproducibility with two widely used intermittent (iTBS) and continuous TBS (cTBS) protocols in a single cohort, and (3) determine the reproducibility of their neuromodulatory effects across identical repeat sessions. We applied 1 Hz, 10 Hz, iTBS, cTBS and sham-rTMS to the hand region of the left motor cortex (M1), where the neuromodulatory effects of rTMS are typically studied. We measured peak-to-peak MEP amplitude in response to single-pulse TMS (spTMS), as it is the most commonly used metric in both experimental and clinical TMS research, to index immediate (5 min post rTMS) and delayed (25 min) changes in corticospinal excitability following each rTMS protocol. Furthermore, as MEP responses are reported to be sensitive to a collection of methodological and individual factors [17], we performed a series of control analyses to examine whether (1) coil position deviations from the motor hotspot, (2) the number of MEP trials in a given block, and (3) the magnitude of baseline (pre-rTMS) corticospinal excitability affect neuromodulatory rTMS responses and their reproducibility.

## Methods

### Participants

Data from 28 healthy participants (10 females, 39 ± 16 years old) across 10 sessions for each participant (280 sessions) were analyzed. All participants met the inclusion criteria, including no known history of psychiatric or neurological diseases, no systemic illness, no contraindications to TMS or magnetic resonance imaging (MRI), and no drug abuse or dependence as measured by the mini-international neuropsychiatric interview (MINI). All experimental protocols were approved by the Institutional Review Board of the Beth Israel Deaconess Medical Center (Boston, MA) and explained to participants while obtaining written informed consent.

### Data acquisition

Real and sham TMS were applied with a 75-mm (outer wing diameter) MagPro Cool B-65 coil and A/P Cool-B65 coil, respectively, attached to a MagPro X100 stimulator (MagVenture A/S). High-resolution anatomical MRIs were obtained for each subject and used for targeting TMS within and across sessions using the neuronavigation system Brainsight® (Rogue Research Inc). Electromyography (EMG) was recorded from the right first dorsal interosseous (FDI) muscle and digitized using the BrainVision actiCHamp amplifier system and recorder software (Version 1.20.0601, Brain Products GmbH). Ag-AgCl surface electrode pairs were placed in a monopolar fashion over the FDI belly and tendon, with a ground electrode placed on the right ulnar styloid. EMG data were sampled at a rate of 5000 Hz and visualized over a 150 ms window length from 50 ms before to 100 ms after the TMS pulse.

### Experimental procedures

**Fig 1:**
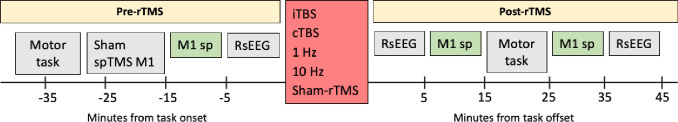
Protocol timeline pre- and post- repetitive transcranial magnetic stimulation (rTMS) application. Baseline motor evoked potential (MEP) amplitudes were obtained by applying 150 single pulses (sp) of TMS to the motor cortex (M1) at 120% resting motor threshold (RMT) and measuring peak-to-peak amplitudes of the elicited MEPs. 150 single pulses were again applied to M1 at 5 (T5) minutes post-rTMS and 60 single pulses delivered at 25 (T25) minutes post-rTMS to assess corticomotor reactivity. Resting state EEG (rsEEG) and behavioural outcomes from a motor task were also obtained before and after rTMS.

This report is a part of a larger TMS-EEG-EMG-behavior study, with the current manuscript focusing on the MEP results to evaluate cortico-spinal excitability (Fig 1). All participants underwent ten separate experimental visits consisting of five rTMS conditions: 1 Hz, 10 Hz, iTBS, cTBS, and sham-rTMS (randomly assigned to one of the four real rTMS protocols) repeated 1 month apart. Sham-rTMS was randomly selected among the four real rTMS protocols for each participant (1 Hz, 10 Hz, iTBS, cTBS) to account for non-specific effects of the experimental procedure on cortical excitability measures. Sham coils generated a similar sound to that of the real coil, and surface-stimulating electrodes were placed above and beside the left eye to simulate somatosensory sensations arising from real TMS. The order of the first five visits was randomized across participants and scheduled at least four days apart to avoid carry-over effects. These visits were then repeated one month later, in the same order for the remaining five visits to evaluate test-retest reliability. Females were tested at approximately the same point in their menstrual cycle and visits were completed at approximately the same time of day for each participant to minimize variability due to circadian fluctuations.

Participants were seated in a chair and wore in-ear headphones with integrated ear-protection throughout all stimulation protocols. Auditory white noise was also used to mask the TMS coil click throughout the stimulation protocols, with the volume set to the maximum level the subject could comfortably tolerate. Following International Federation of Clinical Neurophysiology (IFCN) guidelines, resting motor threshold (RMT) was determined on the FDI hotspot as the minimum stimulation intensity eliciting at least five MEPs (>50 mV) out of ten pulses in the relaxed FDI using biphasic (posterior-anterior in the brain) current waveforms. Active motor threshold (AMT) was then assessed as the minimum stimulus intensity that produced a MEP of at least 200 μV by a cortical silent period in at least five of the 10 trials while subjects were asked to abduct their index finger towards their thumb to slightly contract their FDI muscle. The intensity of spTMS for pre- and post-rTMS corticospinal excitability measurements was set to 120% of RMT. Baseline (pre-rTMS) corticospinal excitability was assessed by applying 150 spTMS trials with randomly jittered (3 to 5 s) inter-stimulus intervals to M1 during the relaxation of the FDI muscle and measuring peak-to-peak amplitudes of the elicited MEPs. Corticospinal excitability was reassessed at 5 (T5) and 25 (T25) minutes following real or sham rTMS with 150 and 60 spTMS trials, respectively. A sequential finger tapping task [18] was also performed at the beginning of each session and 15 min post-rTMS (Fig 1). Participants were asked to press a five-element sequence (such as 4-1-3-2-4) using their index, middle, ring and little finger. By applying novel sequences in each block, we aimed to minimize transfer and learning effects between blocks.

The intensity of rTMS protocols was set based on a prior study evaluating the efficacy of rTMS effects at different TMS intensities [19]. 10 Hz rTMS was applied at 120% RMT in 4-second trains, with a 26-second inter-train interval for 37.5 minutes (3000 pulses total). 1 Hz rTMS was applied at 110% RMT as a continuous train for 15 minutes (900 pulses total). iTBS and cTBS were applied at 80% AMT in three-pulse bursts at 50 Hz with a 200-ms inter-burst interval. For iTBS, this pattern was delivered in two-second trains with an 8-second inter-train interval for 192 seconds (600 pulses total). For the cTBS protocol, the pattern was applied continuously for 40 seconds (600 pulses total). Finally, sham rTMS/TBS was applied with the shielded side of a MagPro A/P Cool-B65 (MagVenture) coil over the at M1 hotspot with a 3-cm thick plastic spacer to further minimize any residual magnetic field reaching the cortex.

### EMG pre-processing

All collected EMG data was processed offline using customized automated scripts running in Matlab R2021a (Math-Works Inc., USA). EMG data were bandpass filtered at 10–2000 Hz and baseline corrected by subtracting the mean value from 50 to 5 ms pre-TMS stimulation from the entire elicited signal. Next, the root mean square (RMS) of the EMG signal from -20 ms pre-TMS pulse to 13 ms post-TMS pulse, omitting -2 to +2 ms to avoid pulse artifact, was calculated to identify trials with artifacts, such as concurrent muscle activity. Trials with RMS values greater than 2.5 standard deviations (SD) from the average RMS of the entire block of trials were removed. On average, 2.1 ± 0.9% of the trials across all participants were more than 2.5 SD from the mean. Peak-to-peak MEP amplitudes were then calculated for each remaining trial as the peak-to-peak voltage from 18 ms to 50 ms post-TMS.

### Statistical analyses

The following statistical analyses were performed using a two-tailed 95% confidence interval. Data were tested for normality using the Shapiro-Wilk test and all non-normal data were log-transformed. P-values for individual tests were Bonferroni-corrected to minimize type-I error.

For the neuromodulatory effects of rTMS, our goal was to test for a main effect of *Time* (Baseline, T5 and T25) for individual rTMS protocols in the initial (test) Visit 1 (V1) and, if so, whether we can reproduce these effects in the retest Visit 2 (V2) with an independent set of analyses. Thus, mean MEP amplitudes from each spTMS block for each condition (1 Hz, 10 Hz, iTBS, cTBS, Sham) and visit (V1 and V2) were entered into separate linear mixed-effects models (LMMs) to examine the main effect of *Time*.

To compare MEP changes from each real rTMS protocol to sham-rTMS, we calculated the ratio of mean MEP amplitudes at post-rTMS time points (T5 and T25) to baseline for each protocol. These ratio values for sham-rTMS at T5 and T25 were subtracted from real rTMS (1 Hz, 10 Hz, iTBS, cTBS) ratio values at T5 and T25 at the subject level. These sham-subtracted ratios were entered into separate mixed-effects linear regression models for each real rTMS condition and visit. A sham-subtracted ratio of greater than zero implies facilitation induced by the real rTMS condition relative to sham, whereas a sham-subtracted ratio of less than zero implies inhibition.

To test whether neuromodulatory effects differ across rTMS protocols, post-rTMS ratio values from each protocol (including sham-rTMS) were entered into a LMM with *Protocol* (1 Hz, 10 Hz, iTBS, cTBS, sham-rTMS) and *Visit* (V1 and V2) as main factors and “Protocol*Visit” as the interaction factor.

### Power analyses

We performed post-hoc analyses for estimating statistical power to detect significant rTMS effects in our sample (n = 28) using previously reported effect sizes. Chung et al. [20] found that iTBS resulted in significant and moderately large MEP increase at T5 and T25 with a pooled effect size (SMD) of 0.69 and 0.71 (Cohen’s D), respectively. They also reported significant and large cTBS effects with -0.9 and -0.69 at T5 and T25 post-cTBS measurements, respectively. We used separate linear mixed-effect model (LMMs) to examine the main effect of Time (Baseline vs T5 vs T25) for each rTMS protocol. However, as power calculations for LMMs are complex and difficult to implement [21], we performed repeated-measures ANOVA as a proxy of LMMs. With a sample size of 28 and an alpha of 0.05, we will have 80% power to detect changes with an effect size (Cohen’s f) of 0.29. For post-hoc t-test comparisons, with three comparisons and a Bonferroni-corrected alpha of 0.0167, we will have 80% power to detect changes with an effect size (Cohen’s d) of 0.57 (a moderate effect). We noted that these effects sizes are considerably lower than what has been reported in the meta-analyses of Chung et al. [20], suggesting that our study is sufficiently powered to detect even smaller effects of rTMS on cortico-spinal excitability.

### Control analyses to examine potential sources of low reliability

To ensure that coil deviations from the motor hotspot and cumulative trend effects due to the high number of MEP trials did not confound the neuromodulatory effects of rTMS, we first examined whether the high number of MEP trials at baseline and T5 blocks induces changes in M1 excitability (See Figure S1 in S1 File). We then ran a series of exploratory analyses looking at coil handling/position and trial hysteresis as potential sources of variance. Specifically, we reran the mixed-effects linear regression models for the neuromodulatory effects of rTMS (i.e., a model with a main effect of *Time*) using subsets of the data by selecting 25 MEP trials that are closest to the motor spot based on Euclidian distance measures, and the first 25 MEP trials regardless of the coil position for each spTMS block, respectively.

At least 25 trials were included in each block for all mean MEP assessments [17]. The group-averaged number of MEP trials across all rTMS sessions were following; 140±18.07, 127±17.55 and 58±1.30 for visit-1; and 141±18.08, 133±14.86 and 59±1.06 for visit-2 within baseline (Pre) and post rTMS blocks (T5 and T25), respectively. In total, 11/840 (1.3%) of the blocks were discarded due to noisy and artifactual trials, 15/840 (1.8%) due to a scaling issue in the data, and 8/840 (1.0%) due to noisy data. Further, for the closest 25 analyses, 62/840 (7.3%) of the data were lost because of triggers not properly sending between brain vision and the EMG recording software. In total, 4% of the data were lost for analyses involving all trials and the first 25 trials, and 11% were lost for the closest 25 trial analyses.

Additionally, to examine the impact of the inter-individual difference in baseline excitability, each visit for each participant was classified as either high or low excitability, depending on whether the average baseline MEP value of a given participant visit was above or below the median baseline MEP value (0.714 mV) across all visits and participants. Baseline excitability groups (High and Low) were then included in a mixed-effects linear regression model as a main factor with Time and Visit to assess for any main or interaction effects in a separate mixed-effect analysis using all MEP trials.

Finally, reproducibility of rTMS effects, including the control analyses, were assessed using interclass correlation coefficients (ICCs). ICC values < 0.25 indicate little to no reliability, 0.25 to 0.5 reflect low reliability, 0.5 to 0.75 indicate moderate reliability, and > 0.75 indicate high-reliability [22, 23].

## Results

### Neuromodulatory rTMS effects

**Fig 2:**
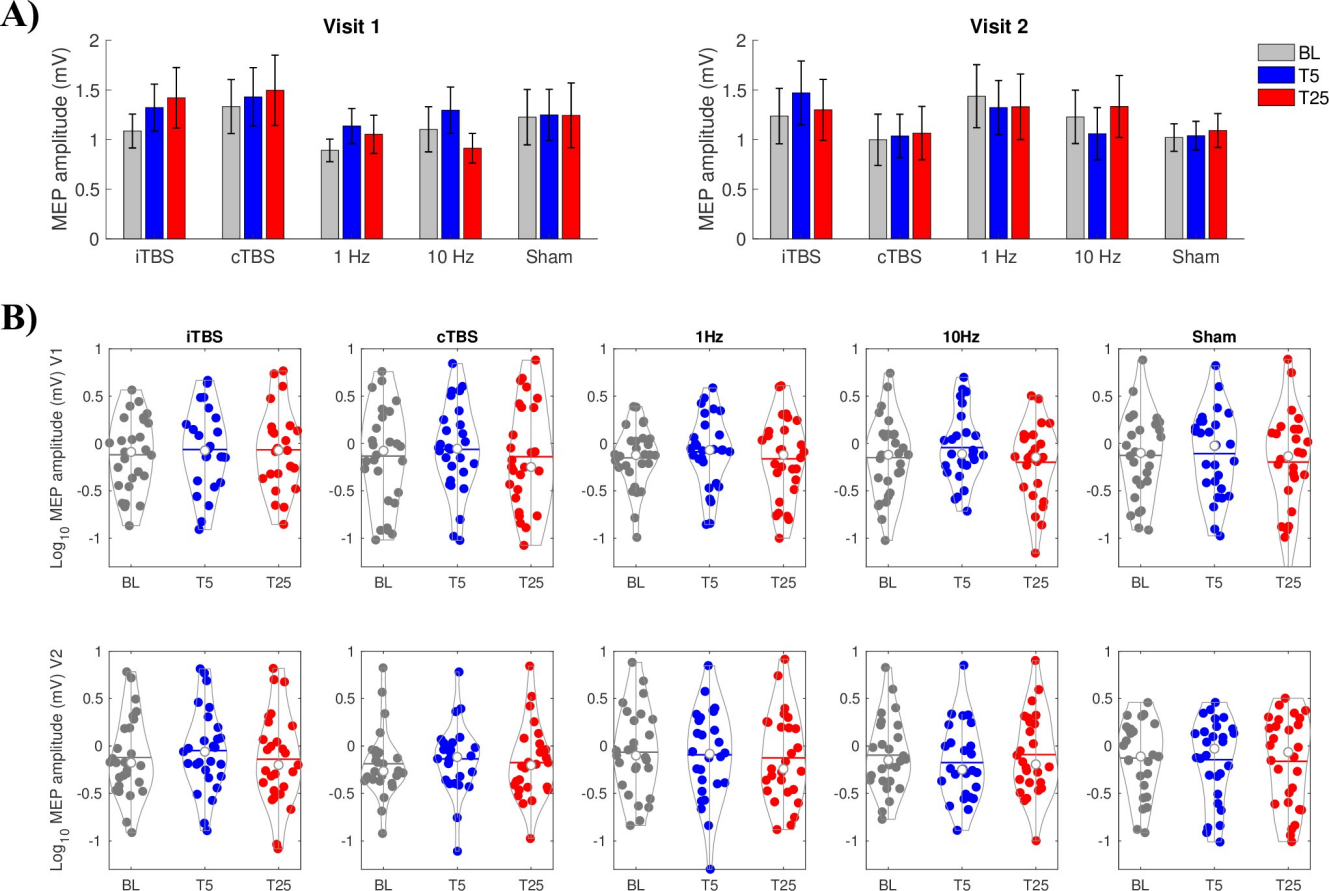
Repetitive transcranial magnetic stimulation (rTMS) protocol effectiveness and repeatability for all motor evoked potential (MEP) trials in each block. (A) Group-averaged MEP at baseline, T5 and T25 during Visit 1 (left) and Visit 2 (right) for intermittent theta burst stimulation (iTBS), continuous theta burst stimulation (cTBS), 1 Hz, 10 Hz, and Sham protocols. Data are mean ± standard error. (B) Individual participant data for Visit 1 (top, V1) and Visit 2 (bottom, V2) for each protocol. White dots show group means.

Group averaged and log transformed MEP amplitudes for each participant at each block (Baseline, T5, T25) across rTMS conditions and visits are shown in Fig 2A and 2B. For each rTMS protocol and visit, we tested for a main effect of *Time* (Baseline, T5 and T25). Across all trials, no statistically significant main effect of *Time* was found for any rTMS protocol (*p* ≥ 0.0558), except for 10 Hz in Visit 2 (F(2,50) = 3.959, p = 0.0253, η^2^ = 0.137) (Fig 2A). However, this result was no longer significant after correction for multiple comparisons.

### Sham-corrected rTMS effects

**Fig 3:**
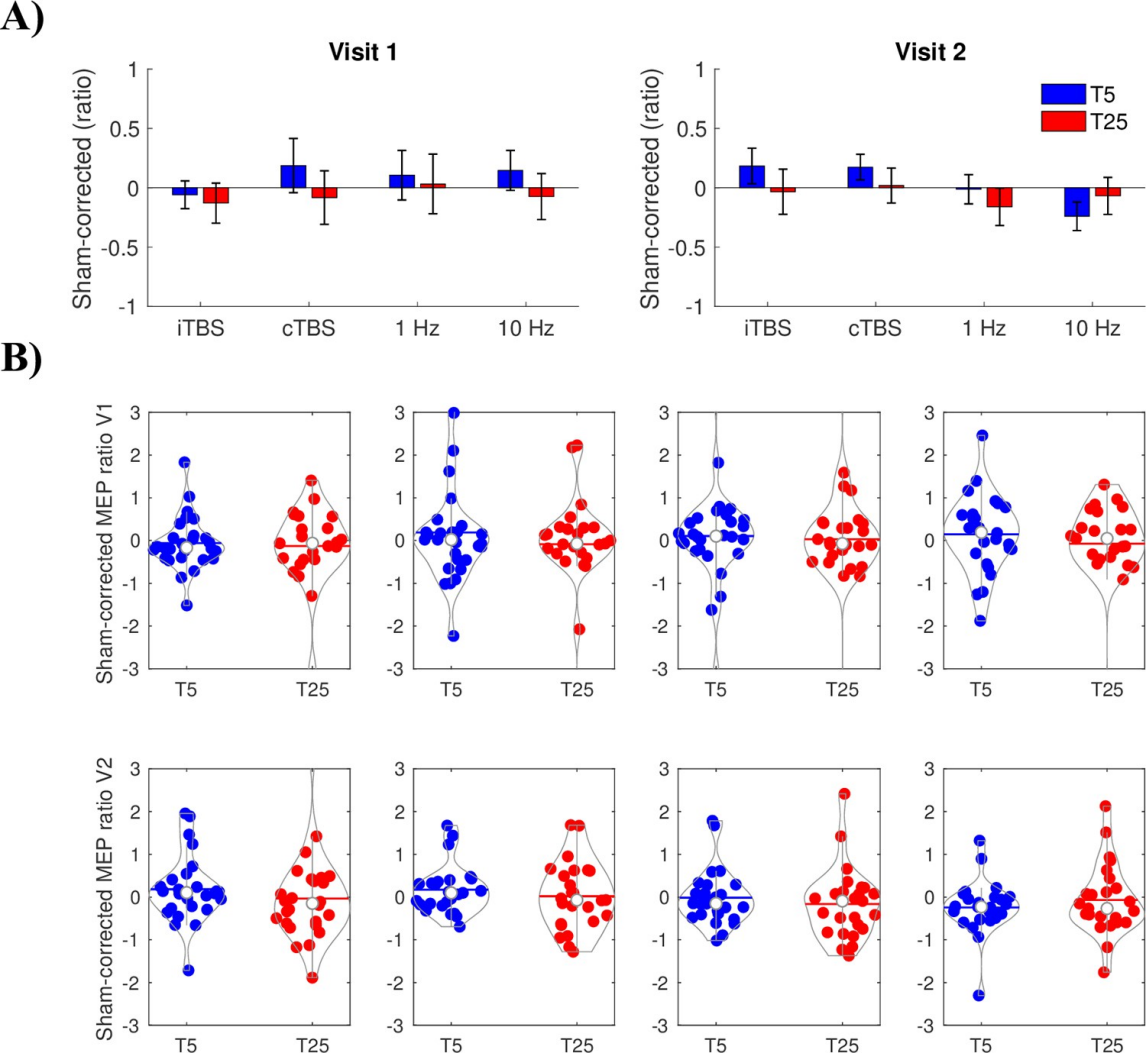
Sham-corrected effects of intermittent theta burst stimulation (iTBS), continuous theta burst stimulation (cTBS), 1 Hz and 10 Hz. (A) Group-averaged motor evoked potential (MEP) ratio values for visit 1 and visit 2 with sham subtracted out separately for each participant. Data are mean ± standard error. (B) Individual sham-corrected data for visit 1 (V1) and visit 2 (V2) for each repetitive transcranial magnetic stimulation (rTMS) protocol. Data are presented as MEP ratio for each rTMS protocol with sham MEP percent change subtracted from these effects separately for each participant.

No main effect of Time was statistically significant for any of the rTMS protocol visits (*p* = 0.391, Fig 3A and 3B). These results indicate low rTMS modulatory effectiveness when accounting for sham.

### Neuromodulatory effect differences among rTMS protocols across visits

The mixed models found that MEP ratios (Post-iTBS/Baseline) at T5 showed a main effect of *Visit* (F(1,28) = 4.479, *p* = 0.0435, η^2^ = 0.138), however, this effect was no longer significant after correction for multiple comparisons. No other main effect or interactions were statistically significant for T5 or T25 ratios (*p* ≥ 0.291).

### rTMS effects when accounting for baseline excitability, the distance of TMS to the hotspot, and the number of trials

**Fig 4:**
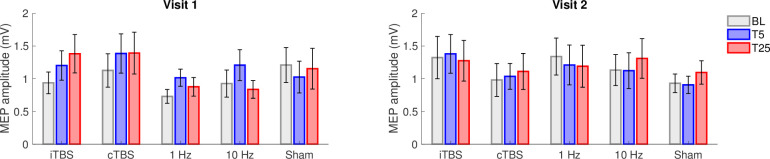
Repetitive transcranial magnetic stimulation (rTMS) protocol effectiveness and reproducibility for the first 25 motor evoked potential (MEP) trials in each block. Group-averaged MEP at baseline (BL), T5 and T25 during Visit 1 (V1; left) and Visit 2 (V2; right) for intermittent theta burst stimulation (iTBS), continuous theta burst stimulation (cTBS), 1 Hz, 10 Hz, and Sham protocols. Data are mean ± standard error.

**Fig 5:**
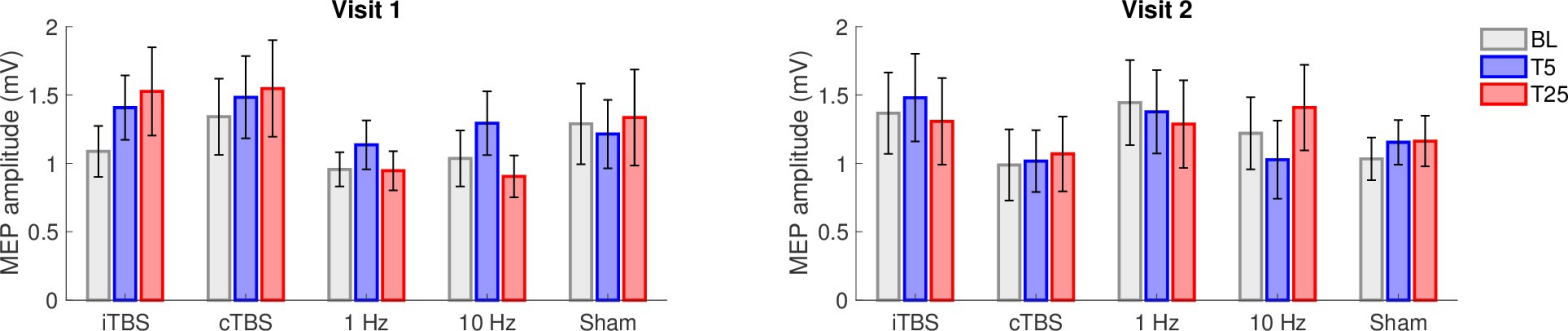
Repetitive transcranial magnetic stimulation (rTMS) protocol effectiveness and repeatability for the 25 trials corresponding to the single pulse stimulation applied closest to the hotspot. Group-averaged motor evoked potential (MEP) at baseline, T5 and T25 during visit 1 (left) and visit 2 (right) for intermittent theta burst stimulation (iTBS), continuous theta burst stimulation (cTBS), 1 Hz, 10 Hz, and Sham protocols. Data are mean ± standard error.

Baseline excitability, the distance of the TMS coil to the hotspot, and the number of trials were all included in the mixed model assessing a main effect of time to determine whether these factors would account for any MEP amplitude changes in response to the various rTMS protocols. rTMS effects were not improved when accounting for only the first 25 trials as reflected by the lack of statistical significance for the main effect of Time for these data (*p* = 0.0643) (Fig 4) except for 1 Hz (F(2,54) = 3.451, *p* = 0.0389, η^2^ = 0.113) and iTBS in visit 1 (F(2,46) = 3.425, *p* = 0.0411, η^2^ = 0.130). However, when correcting for multiple comparisons (p > 0.005), these results were no longer significant, and consistent effects were not present in Visit 2. When accounting for the closest 25 trials to the motor hot spot only, a significant main effect of Time was found for 10 Hz in visit 2 (F(2,46) = 5.789, *p* = 0.0057, η^2^ = 0.201), however, again this finding did not survive multiple comparisons (Fig 5). No other main effect of Time was found for the closest 25 trials (*p* ≥ 0.0843).

**Fig 6:**
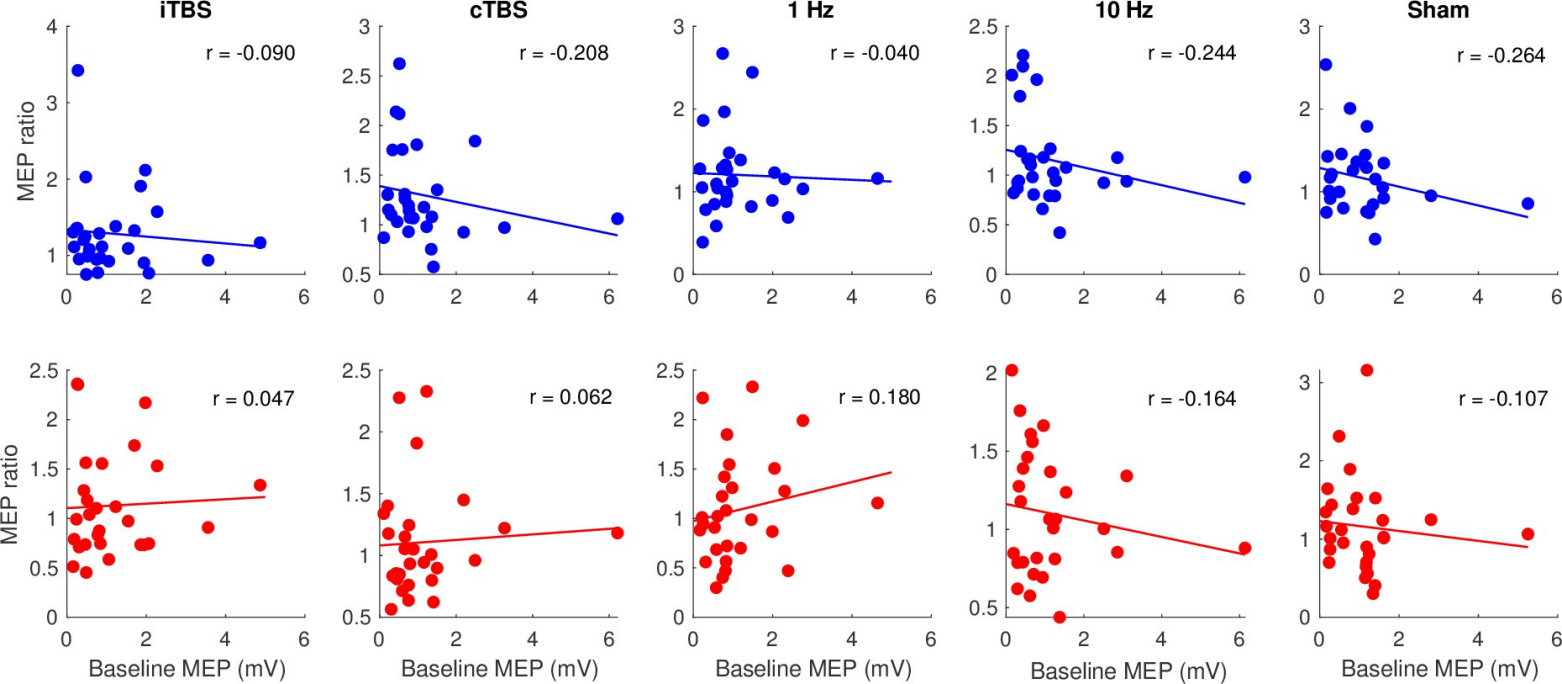
Scatter plots between baseline motor evoked potential (MEP) amplitude and post repetitive transcranial magnetic stimulation (rTMS) MEPs as a percent of baseline averaged across visits for T5 (top) and T25 (bottom) for each protocol; dots represent individual participants.

For the baseline excitability assessment, a significant main effect of Baseline Excitability was found for all protocols (p < 0.0001), confirming that the high and low baseline excitability groups are significantly different from each other (Fig 6). A significant interaction between Time and Baseline excitability was found for both the 1 Hz (F(2,85) = 3.255, *p* = 0.0434, η^2^ = 0.071) and cTBS (F(2,102) = 5.515, *p* = 0.0053, η^2^ = 0.098), however, only the cTBS protocol effect remained significant after correction for multiple comparisons. Post-hoc analyses showed that the low baseline MEP group had a significant increase in MEP amplitude after cTBS at T5 compared to baseline (t = -3.582, *p* = 0.0006). Notably, this effect was only significant for Visit 1 and was not reproduced independently in Visit 2. No significant main effects or interactions were found for any of the other protocols (*p* = 0.142).

### Reproducibility of neuromodulatory rTMS effects

**Fig 7:**
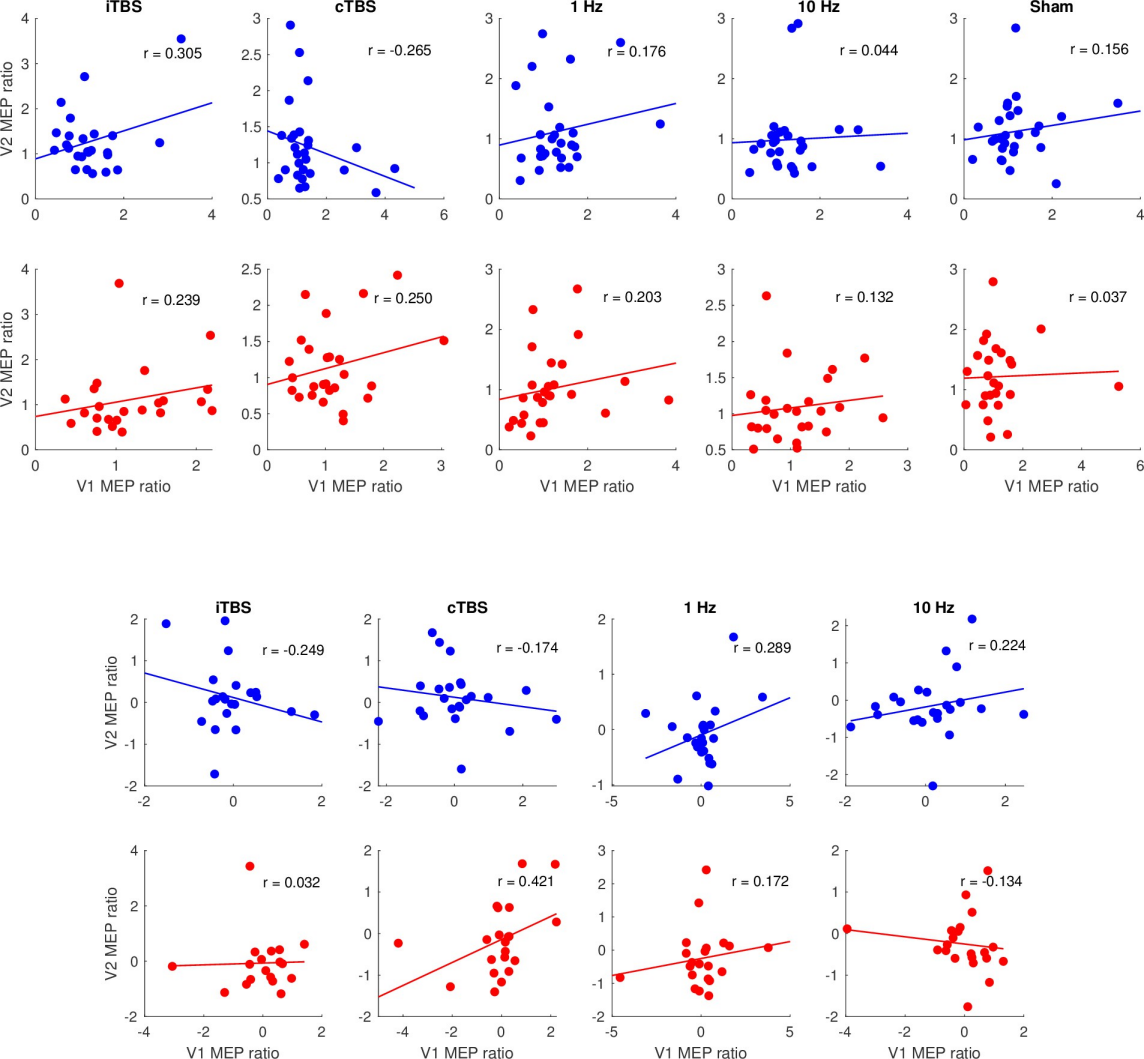
Scatter plots between T5 (blue) and T25 (red) ratio data during visit 1 (V1) and visit 2 (V2) for each protocol with a single dot representing each participant for (A) all trials and (B) sham-corrected data.

The reliability of treatment effects was measured using intraclass correlation coefficients. Scatter plots showing a correlation between visit 1 and visit 2 for the ratio data at T5 and T25 are presented in Fig 7A for all trials and 7B for sham-corrected trials. ICC results for MEP ratios at T5 and T25 for all protocols are shown in Table 1. Each protocol time point showed poor reliability across visits when using all trials to calculate T5 and T25 ratio values. Poor reliability was also found for the T5 and T25 ratio values based on the first 25 trials only. After correcting for multiple comparisons, only the sham T5 closest 25 trials showed moderate reliability across visits (c = 0.624, *p* = 0.002). Baseline excitability was also moderately reliable across all 10 visits (c = 0.5774, *p* < 0.001).

**Table 1:** Intra-participant variability results showing c and p-values for the intraclass correlation coefficient (ICC) between visit 1 and visit 2 using ratio data at T5 and T25 for all trials, first 25 trials, closest 25 trials and sham-corrected data. P-values less than 0.05 are highlighted in light gray, and p-values less than 0.005 (surviving multiple comparisons) are highlighted in dark gray.

	All trials		First 25		Closest 25		Sham-corrected	
	**r-value**	**p-value**	**r-value**	**p-value**	**r-value**	**p-value**	**r-value**	**p-value**
iTBS T5	0.323	0.051	0.298	0.067	0.595	0.004	-0.255	0.892
iTBS T25	0.247	0.118	0.460	0.011	0.397	0.043	0.056	0.403
cTBS T5	-0.224	0.875	0.092	0.319	-0.219	0.838	-0.127	0.737
cTBS T25	0.266	0.087	0.152	0.221	0.087	0.350	0.378	0.032
1 Hz T5	0.169	0.192	-0.115	0.721	-0.005	0.508	0.216	0.137
1 Hz T25	0.192	0.165	0.079	0.345	0.183	0.203	0.151	0.238
10 Hz T5	-0.016	0.531	0.248	0.099	0.001	0.498	0.198	0.158
10 Hz T25	0.149	0.231	-0.080	0.651	0.407	0.031	-0.109	0.690
Sham T5	0.166	0.196	0.040	0.418	0.417	0.035	-	-
Sham T25	0.052	0.397	0.129	0.262	-0.190	0.761	-	-

### Descriptive classification of corticospinal responses to rTMS protocols

**Fig 8:**
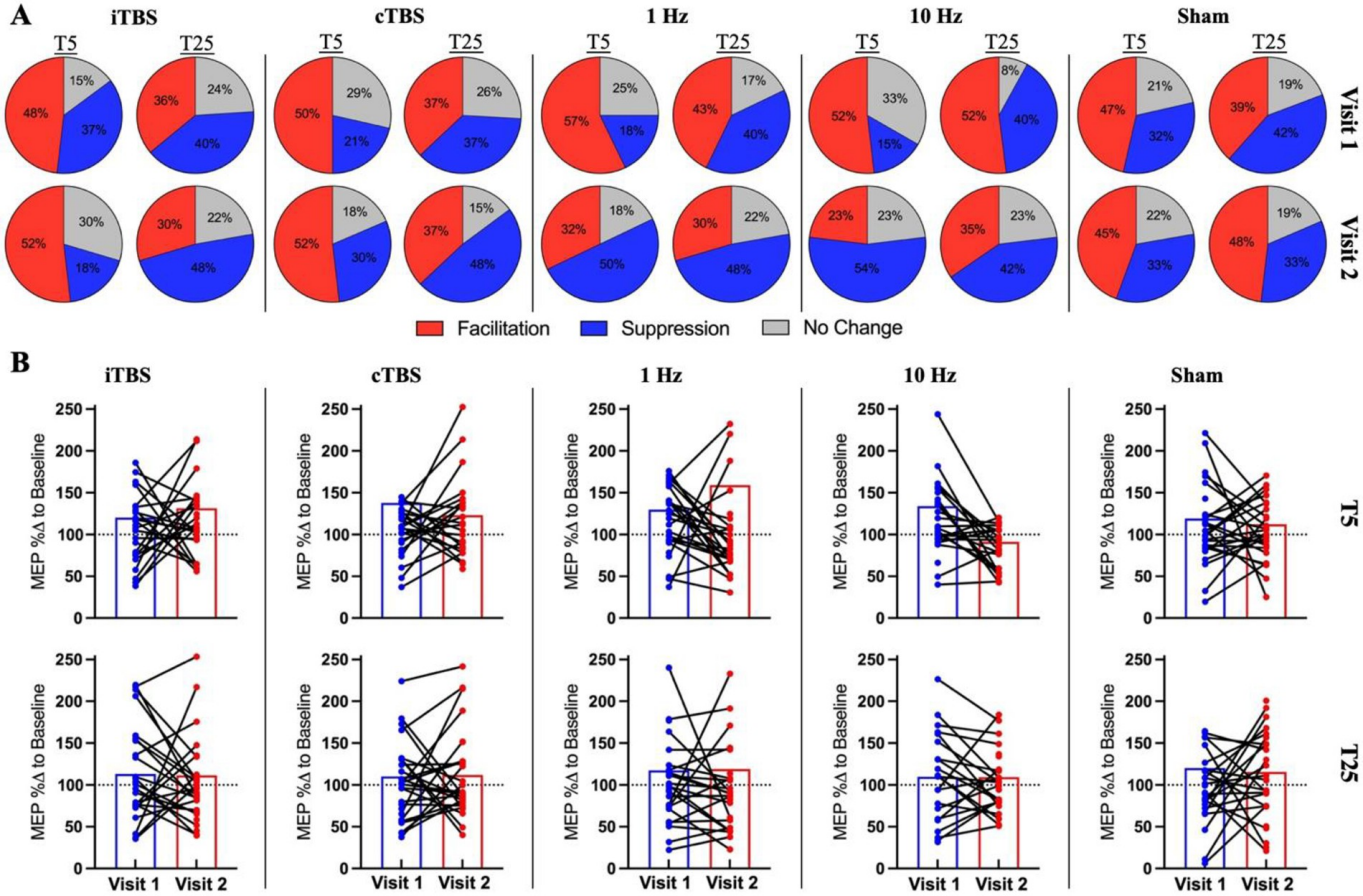
Individual response characteristics to rTMS. **(A)** Classification of individual responses and responder types across rTMS protocols for each visit. Pie charts show the percentage of facilitation (Red: μ≥110%), suppression (Blue: μ≤ 90%) and no change (Gray: 110%<μ>90%) for each rTMS protocol in Visit-1 (upper pie charts) and Visit-2 (Lower pie charts). **(B)** Bar graphs showing group averaged MEP percentage changes across visits (Visit 1 and Visit) and blocks (T5 and T25). The colored dots (blue for Visit 1 and red for Visit 2) denotes the individual response and the black lines connected to the dots represent and track individual response changes across visits (MEP %Δ to Baseline; motor evoked potential percentage change to baseline).

We classified rTMS response types using the percentage change in MEPs from baseline, as previously described [24]. In particular, facilitation response is defined as an increase of at least 10% from baseline (μ≥110%), suppression response is defined as a decrease of at least 10% from baseline (μ≤90%) and others within these ranges (110%<μ>90%) is defined as no change/responder. At the group level, we did not see expected dominant pattern in any of the rTMS protocols including sham. For example, for iTBS we observed that roughly half of the participants (48% in Visit-1 and 52% in Visit-2) showed the expected facilitatory responses at T5 for both visits, but these responder ratios were very similar to the ratio of facilitatory responses following sham stimulation (47% in Visit-1 and 45% in Visit-2). Moreover, individual response patterns were also not consistent across visits such that a given person classified as responder to iTBS with increased MEPs in Visit-1 may show opposite or no-response to the same stimulation in Visit-2 (Please see Fig 8A). Similar, high variability in intra-individual responses was observed for all rTMS blocks.

## Discussion

In this study, we assessed whether 1 Hz and 10 Hz rTMS protocols (1) significantly decrease or increase corticospinal excitability, respectively, against sham-rTMS, (2) differ from widely used TBS protocols (iTBS and cTBS) on modulating corticospinal excitability, and (3) whether effects of both traditional rTMS and TBS protocols on corticospinal excitability are reproducible across identical sessions. We found that neither 1 Hz nor 10 Hz rTMS had any significant effect on modulating corticospinal excitability at the group level, as indexed by the amplitude of MEP responses. MEP responses to these protocols were also not significantly different from both sham-rTMS and two widely used TBS protocols. Reproducibility of the neuromodulatory effects was poor for both real rTMS and TBS protocols except for iTBS at T5 for a subset of MEP trials (*n* = 25) when coil position was closest to the stimulation target. Importantly, none of the real rTMS protocols demonstrated consistent corticospinal neuromodulation or reproducibility after controlling for baseline corticospinal excitability, TMS coil deviation and number of individual MEP trials within a given block. These results, along with the recent reports showing substantial variability and poor replicability in TBS applications [9, 10, 12, 14, 16, 25] call for a fundamental rethinking of the potential mechanisms by which rTMS affects brain function and behavior in humans.

Recently, we have witnessed a reproducibility/replication crisis in almost every field of brain research including psychology [26], neuroimaging [27–30] and genomics [31], and research in non-invasive modulation of brain function in humans is no exception. Pascual-Leone and colleagues [3], performed the first experimental rTMS study on the human motor cortex and showed increased MEP amplitudes following 10 Hz rTMS of M1, and Wassermann and colleagues [32] reported diminished MEP amplitudes for the first time following rTMS of M1 at 1 Hz. Similarly, the first study [6] assessing the neuromodulatory effects of TBS protocols reported increased and decreased MEP amplitudes following iTBS and cTBS of the human motor cortex, respectively. Following these initial results, rTMS and TBS protocols have been broadly employed as non-invasive therapeutic tools to induce persistent plasticity in targeted brain networks for the treatment of a wide range of neurological [5, 20, 33] and psychiatric [34–36] disorders. These protocols are also popular neuromodulation techniques to promote transient changes in neural excitability and thereby modulate human behavior and cognition in single-session experimental designs [5, 37]. Randomized clinical trials employing multi-session rTMS interventions have provided accumulating evidence for the therapeutic benefits of these rTMS and TBS protocols, resulting in Food and Drug Administration (FDA) approved therapies in major depressive disorder (MDD) [38–40], obsessive-compulsive disorder (OCD) [41, 42] and migraine [43, 44]. Yet, an increasing number of experimental research applying single-session rTMS [24, 45] and TBS protocols [10, 25] in humans have failed to show effective and reliable modulation of neuronal excitability, which is presumed to be the primary neurophysiological mechanism underlying the clinical effects of rTMS.

Previous studies assessing neuromodulatory effects of TBS in humans reported high inter- and intra-individual variability in corticospinal and cortical excitability following TBS [9], and with poor reproducibility of neuromodulatory effects in repeat sessions [12, 22]. Similarly, an early-dated review of 1 Hz and 10 Hz protocols on corticospinal excitability [5] also showed that only less than half of the studies reported expected changes with increased excitability following 10 Hz and decreased excitability following 1 Hz. Unfortunately, however, none of the studies reporting expected rTMS changes [4, 45–53] have included a robust sham protocol to control for placebo or non-specific effects of rTMS, or performed repeat sessions to test the consistency of reported rTMS effects. It is important to note that we could also reach similar conclusions in the current study if we would remove sham comparisons and repeat tests from the statistical analyses. For example, iTBS increased MEPs from baseline to T25 in Visit-1 but this effect was not significant compared to the sham and was not reproduced in Visit-2 (Fig 2). Overall, we have demonstrated that it is not possible to draw valid conclusions regarding the effects of TBS protocols on corticospinal and cortical excitability without having sham-controls and performing repeat tests.

At this point, it is critical to question why a growing number of experimental reports have failed to identify the cortical excitability modulations that are presumed to underlie rTMS effects, while evidence for the clinical efficacy of repeated sessions of rTMS is only growing. The discrepancy between clinical and experimental rTMS studies could be explained by a number of hypothetical factors including, but not limited to, measurement tools and metrics for examining rTMS effects on brain function, methodological differences between clinical and experimental studies, and alternative mechanisms of action for clinical rTMS effects that are yet to be identified. To date, peak-to-peak MEP amplitude has been the most commonly used outcome measure for determining rTMS dose and for assessing the neuromodulatory effects of rTMS applications [54]. Despite such widespread use, MEP amplitudes are known to have substantial variability between successive trials only a few seconds apart, even with constant stimulation parameters and experimental conditions [10, 55–58]. A large body of prior research [17] has demonstrated that variability in MEP amplitudes may be attributed to a collection of experimental and methodological factors including stimulation parameters, devices, stimulated muscles, number of consecutive pulses, millimetric deviations from the hotspot, and the momentary fluctuations in brain state at the time of stimulation. In this regard, we performed a series of control analyses by re-grouping subjects based on their pre-rTMS MEP amplitudes, selecting a subset of MEPs when the coil was closest to the motor hotspot, as well as analyzing only the first 25 MEP trials, to account for potential experimental confounds such as baseline excitability of the stimulated muscle, variability in coil positioning, and cumulative intra-block effects of single-pulse TMS, respectively. Despite these efforts, however, we did not find the expected TMS-induced modulation of corticospinal excitability, nor improved reproducibility of neuromodulation across sessions, suggesting that these factors are unlikely to have significant contributions to high inter- and intra-individual response variability to rTMS reported in this study. Another possibility is that the MEP amplitude itself, as an outcome measure, is not specific enough to the neurophysiological alterations that are specifically occurring within cortical circuits. As MEP responses to rTMS reflect a combination of changes along the entire motor pathway (i.e., cortical, spinal *and* peripheral excitability), it is possible that endogenous fluctuations in the excitability of spinal motoneurons confound rTMS effects or contribute to their variability. In fact, pharmacological studies [59, 60] in humans showed that blocking N-methyl-D-aspartate receptors (NMDARs), a primary synaptic signaling mechanism underlying long-term potentiation (LTP) or depression (LTD) like plasticity [61], suppressed excitatory and inhibitory aftereffects of iTBS and cTBS, respectively. Importantly, however, NMDAR blockage did not alter resting and active motor thresholds when compared to placebo agents, showing that NMDAR blockage mainly eliminated rTMS-induced neuromodulation but did not affect baseline cortical excitability [59, 60]. These results support the idea that subthreshold rTMS mainly induces NMDA-dependent synaptic changes within intra-cortical circuits [62, 63] and suggest that magnitude of these cortical changes may not be adequately and consistently captured by the MEP amplitudes due to their high inter- and intra-subject variability and susceptibility to confounding fluctuations from subcortical and spinal sources. Alternatively, the lack of change in corticospinal excitability following NMDAR blockage may also indicate that MEP responses to sTMS reflect neural activation mechanisms that are not directly involved in rTMS-induced neuroplasticity mediated by activation of NMDAR. For example, previous research has shown that suprathreshold sTMS of the human motor cortex generates high-frequency descending volleys (~700 Hz) in corticospinal tracts with ISIs around 1.5ms [62, 64–66]. NMDA receptors, however, are known to have slow-evolving kinetics with discharge rates longer than 2ms [66, 67], thus they may act as a low-pass filter and are unlikely to involve in producing high-frequency descending volleys following suprathreshold sTMS due to their slower depolarization rates. If sub-threshold rTMS mainly exerts its’ neuromodulatory effects on slower NMDAR and if high-frequency descending volleys in pyramidal tracts following sTMS are preferentially mediated by faster receptors (~700 Hz), it is then conceivable to assume that the MEP response to sTMS may not even be the target outcome measure to detect NMDA-dependent LTP and LTD induced by rTMS in intra-cortical circuits.

Future studies should perhaps focus on outcome measures that reflect spatial-temporal and oscillatory characteristics of neuronal function directly at the cortical level. Combining TMS with scalp electroencephalography (TMS-EEG) enables assessment of TMS-evoked potentials (TEPs) which, compared to MEPs, are typically considered as direct measures of cortical excitability [68–70]. However, a recent study by our group showed no reliable changes in TEPs following theta burst stimulation [25], supporting the current findings that rTMS is likely not modulating local cortical excitability in the way we currently assume it does. Thus, more work is needed in this field to help identify the mechanisms by which rTMS is achieving its effects. For example, spectral dynamics of spontaneous neural oscillations captured during resting brain state (Rest-EEG) may be sensitive to changes in NMDA-dependent plasticity in humans [71], and could be considered another alternative outcome measure to assess rTMS effects.

Another important potential factor for the current discrepancy between experimental investigations showing a lack of consistent changes in cortical excitability and the validated clinical rTMS effects is that in clinical applications, rTMS is typically applied in multiple sessions over several weeks. In contrast, the majority of experimental studies assessing the effects of rTMS on cortical excitability employ single session pre-post designs and measure neuromodulatory effects before and after rTMS with multiple follow-up blocks up to 60–90 mins [5]. The different patterns of application may result in different mechanisms of action. Research in animal models, for example, has shown that a single bout of high-frequency rTMS changes neuronal activity mainly through modulation of membrane potentials [72], with increased steady state neuronal currents, while daily sessions of rTMS protocols at the same frequency resulted in substantially increased BDNF levels [73]. It is possible that short-lived activity-dependent modulation of synaptic plasticity is highly variable both between and within individuals following single bouts of rTMS, but that multiple sessions of rTMS induce persistent expression of neurotrophic factors [74] that result in more consistent network-level changes and therapeutic effects. Finally, it is possible that the cortical excitability hypothesis itself may be insufficient to explain the observed long-term changes in human brain function following multiple rTMS interventions. Emerging evidence in animal models suggested that, apart from cortical excitability, rTMS induces changes in gene expression, modulation of neurotransmitter release, reduced oxidative stress, inflammation, and activation of neurotrophic factors, which together may contribute to the lasting clinical effects of rTMS [63]. However, it is important to note that none of the molecular mechanisms of rTMS has been rigorously tested and directly validated in humans [63].

One limitation of the current study is the use of many different types of assessments (i.e. EEG, motor task, MEP, etc.) to assess rTMS effectiveness and reliability. Our main goal was to measure multiple electrophysiological and behavioral outcomes to comprehensively examine the neuromodulatory effects of rTMS at different levels. However, this makes it possible that one of the variables influenced another, independent of rTMS protocol effects. For example, it is possible that baseline corticospinal excitability reported in the current study was influenced by the motor task. Importantly, these rTMS protocols are typically used in clinical settings to improve outcomes in individuals with depression, schizophrenia, etc. and, therefore, assessing outcomes in these specific populations would be beneficial. Lastly, although we controlled for caffeine intake, sleep and menstrual cycle by instructing participants to follow their regular caffeine intake/sleep patterns, we did not control for other factors such as physical activity levels, which could have affected inter-participant variability. However, factors affecting intra-participant variability were largely controlled for including testing at the same time of day and the use of neuronavigation to ensure similar TMS coil positioning.

### Conclusions and future directions

Repetitive TMS has become a very popular non-invasive therapeutic tool to attempt to treat a wide variety of neuropsychiatric disorders. Apart from FDA-approved therapies in MDD, OCD and migraine, these rTMS protocols have been investigated as a therapeutic tool for stroke recovery [75], addiction [76, 77], schizophrenia [78], autism [79], post-traumatic stress disorders [80], epilepsy [81], tinnitus [82], chronic pain [83], and memory deficits [33], among many others. Despite this widespread use, however, the mechanisms via which rTMS changes human brain function and achieves therapeutic benefits are not well understood. The predominant hypothesis for the mechanism of rTMS effects has been via modulation of cortical excitability. However, our results (and those of others) suggest that this hypothesis may not be valid, at least the way it is typically measured in humans; rTMS does not produce reliable changes in corticospinal excitability either within or across individuals. As long as the complex biological and neurophysiological mechanisms underlying rTMS effects on human brain function and behavior remain unexplained, individual and experimental factors contributing to substantial inter- and intra-subject response variability to rTMS cannot be adequately controlled for, and thus will stand as a major obstacle for efforts to personalize rTMS to improve its consistency and clinical effectiveness. Future studies are immediately needed that use alternative measurement tools and metrics to directly measure brain responses to rTMS, employ longitudinal research designs to monitor changes in brain function and structure and their relationship to behavioral changes over repeated sessions, and study mechanistic hypotheses to understand the role of individual and experimental parameters on rTMS effects.

## Supporting information

S1 File This file contains all the supporting text and figure. (DOCX)
