# Supplementary material for: Neuromodulatory effects and reproducibility of the most widely used repetitive transcranial magnetic stimulation protocols
Source: PLoS One. 2023 Jun 23;18(6):e0286465. doi: 10.1371/journal.pone.0286465 (PMC10289434; doi:10.1371/journal.pone.0286465)
Supplement: S1 File — (DOCX) [file pone.0286465.s001.docx]

**Supporting Figures & Results**

*Trial-based MEP Changes*

To examine whether the high number of MEP trials induces changes in M1 excitability, we conducted additional statistical analysis comparing the first and the last 25 MEPs trials within a given block. We performed paired t-test (two-tailed) at the group level separately for mean MEP amplitudes of the first and last 25 trials of each individual within each rTMS protocol and sham control, and found no significant differences between the first and last 25 MEP trials within baseline blocks (Visit 1: *p* = 0.1253, *p* = 0.1506, *p* = 0.0648, *p* = 0.1821, *p* = 0.9011; and Visit 2: *p* = 0.6795, *p* = 0.2238, *p* = 0.4235, *p* = 0.6157, *p* = 0.3732 for iTBS, cTBS, 1 Hz, 10 Hz and Sham, respectively) (Fig. S1) suggesting that there was no changing trend in M1 excitability due to high number of TMS trials.

| 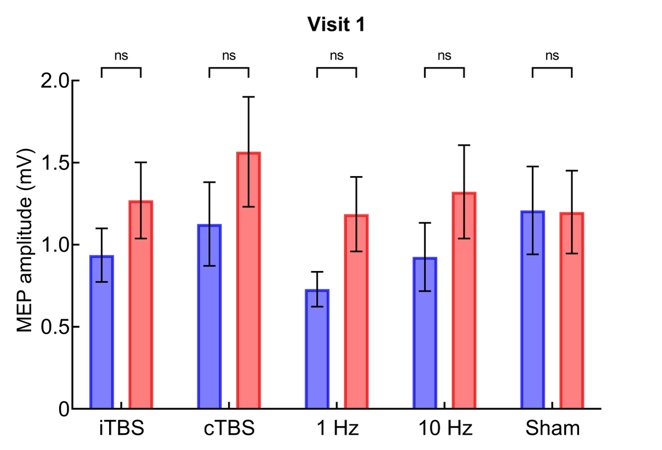 | 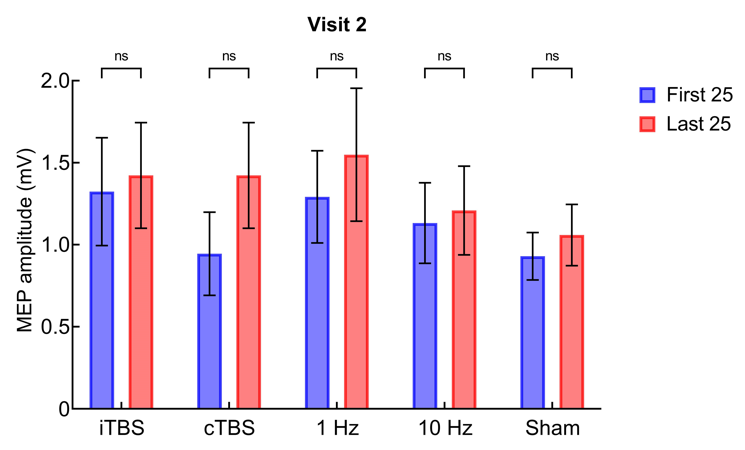 |
| --- | --- |

**Figure S1.** Average MEP amplitudes for the first and last 25 trials within a given block. Group-averaged MEPs at baseline (BL) during Visit 1 (V1; left) and Visit 2 (V2; right) for intermittent theta burst stimulation (iTBS), continuous theta burst stimulation (cTBS), 1 Hz, 10 Hz, and Sham protocols. Data are mean ± standard error.
